# Occipital Hemangiopericytoma 10 Years after Initial Manifestation

**DOI:** 10.5334/jbsr.2099

**Published:** 2020-07-03

**Authors:** Sander Van den Eede, Nick Van de Voorde, Sven Dekeyzer

**Affiliations:** 1 Antwerp University, BE; 2 University Hospital of Antwerp, BE

**Keywords:** Hemangiopericytoma, Solitary Fibrous Tumor, Central nervous system, metachronous, meningioma

## Abstract

**Teaching Point:** Cranial hemangiopericytoma is a rare neoplasm that can be differentiated from meningioma on imaging by its lobulated, mushrooming contours and adjacent osteolysis rather than hyperostosis.

## Case Presentation

**Figure 1:**
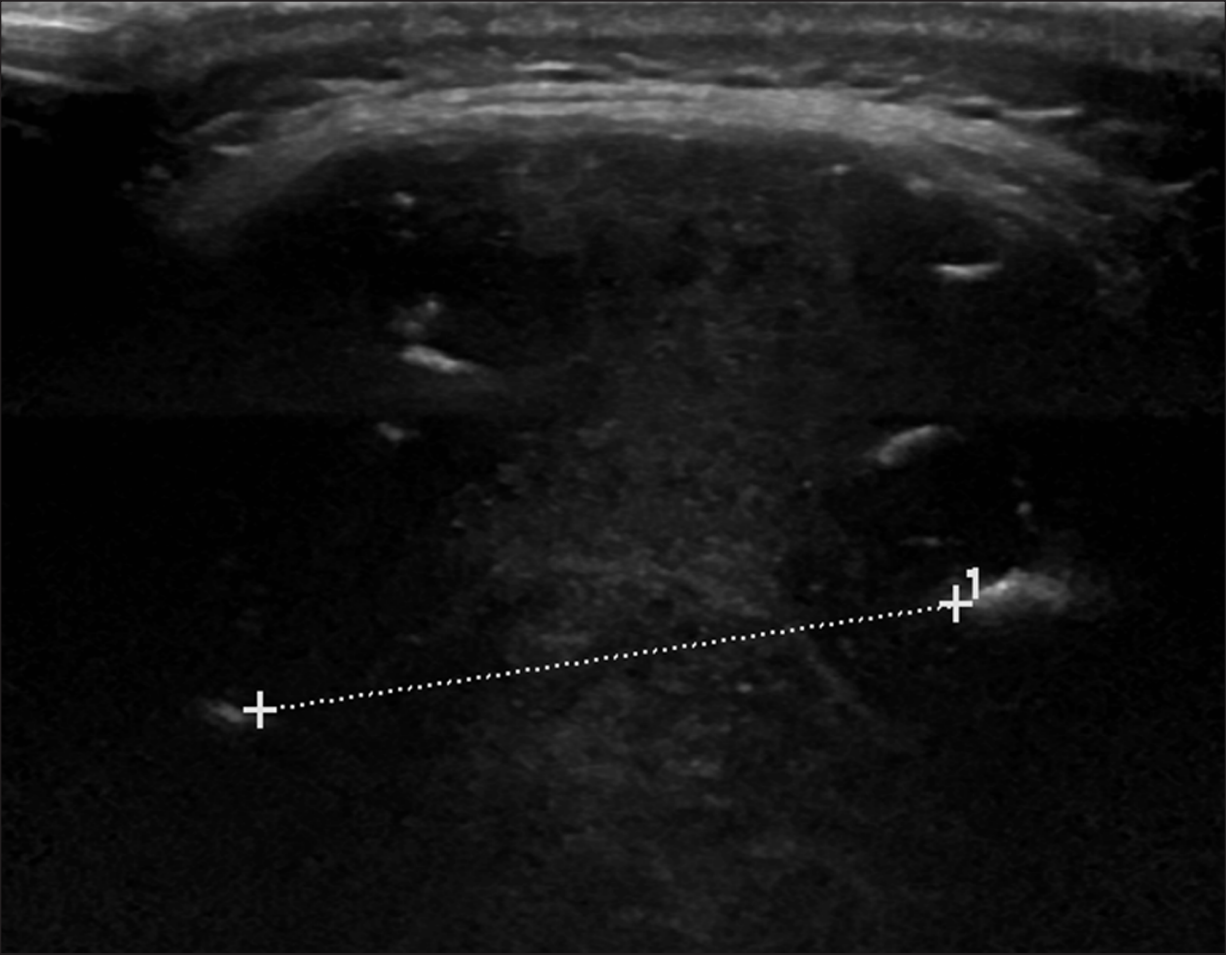
Ultrasound examination of the occipital region shows a heterogeneously hypoechoic mass measuring up to 6 cm.

**Figure 2:**
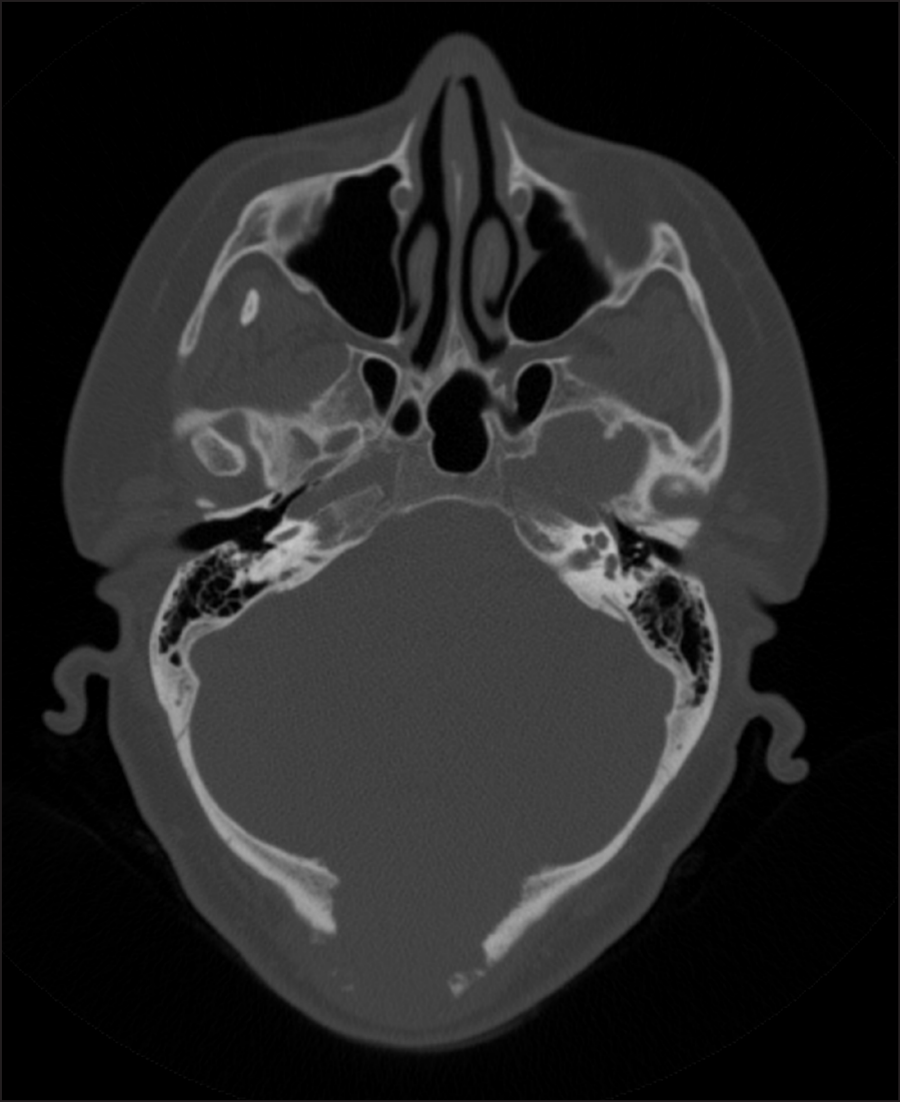
Axial CT reformat (bone window) depicts an extensive osteolysis centrally in the occipital bone with an outward bulging soft tissue mass.

**Figure 3:**
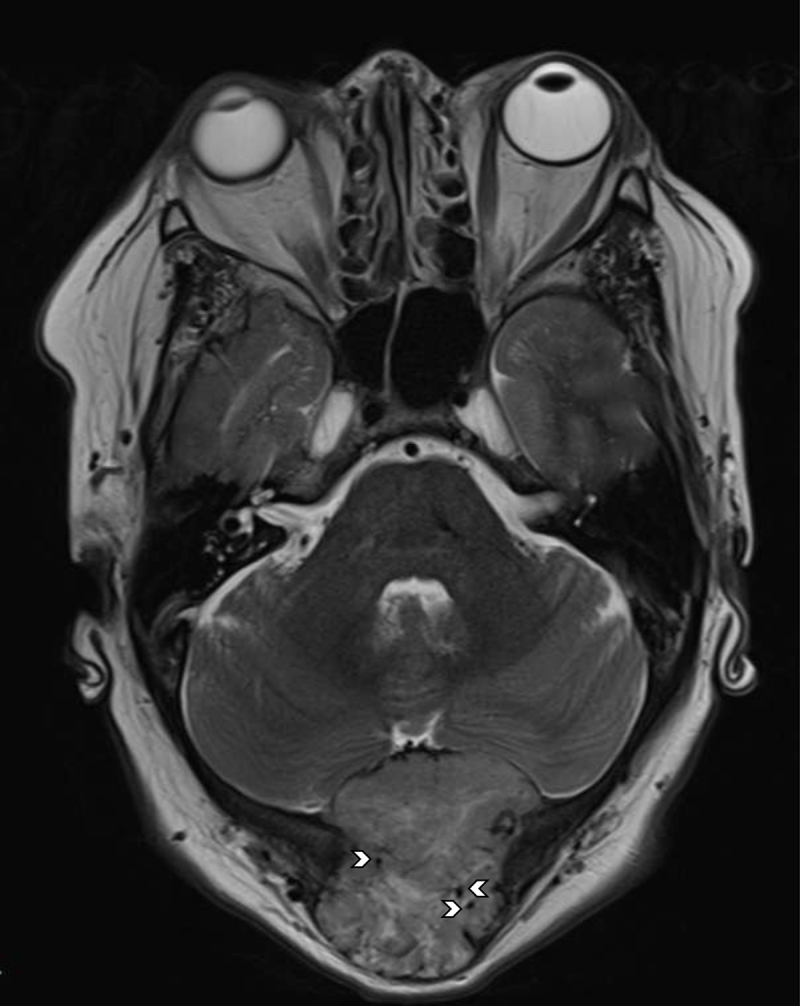
Axial TSE T2-WI shows a heterogeneously iso- to hyperintense lesion centered around the occipital bone. Note the discrete vascular flow voids (arrowheads). Brain edema was absent.

**Figure 4:**
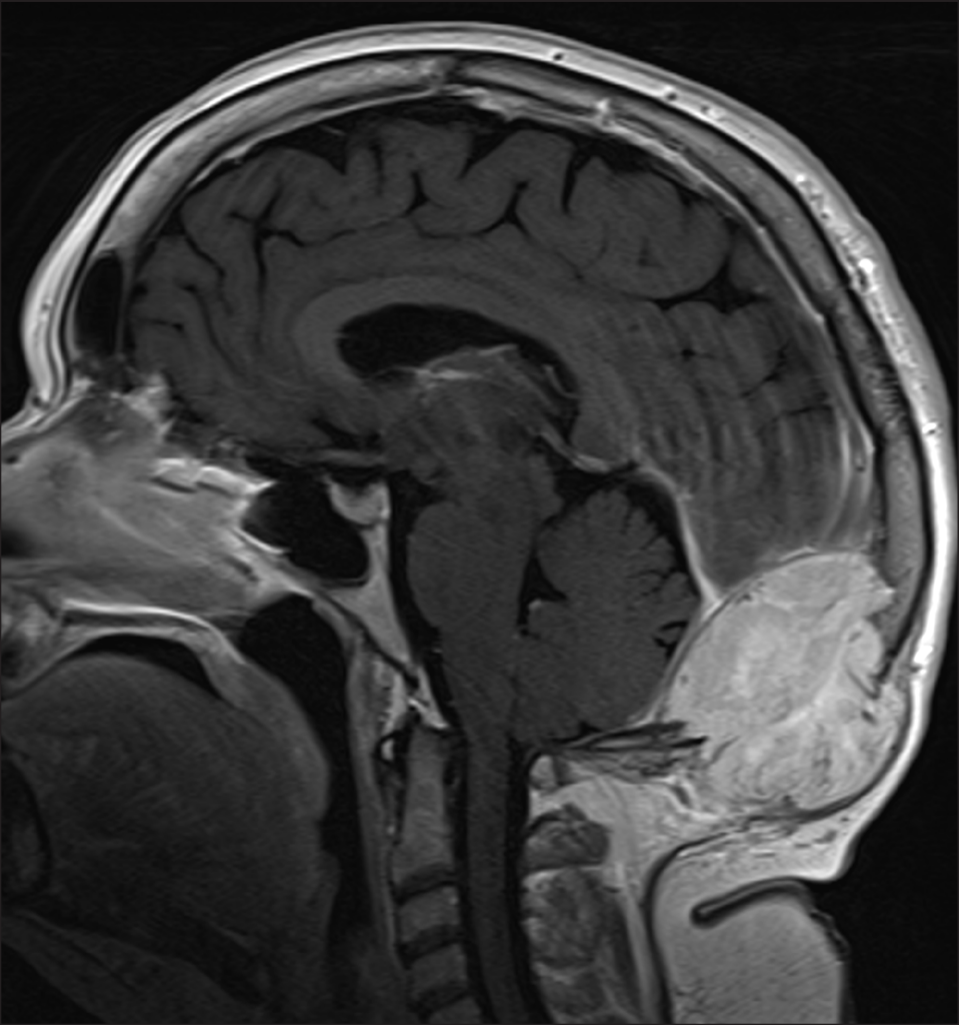
Sagittal T1-WI after injection of gadolinium contrast depicts a vividly enhancing mass with sharply delineated borders.

A 54-year-old woman was referred to our hospital for occipital soft tissue swelling and headache. The patient’s history was remarkable for resection and radiation therapy for a hemangiopericytoma (HPC) in the left gluteal musculature 10 years previously. The patient had also undergone multiple pulmonary metastases resections. An ultrasound of the occipital region revealed a heterogeneous soft tissue mass with destruction of the occipital bone of up to 6 cm (Figure 1). Computed tomography (CT) of the brain showed an extra-axial mass in the fossa posterior extending through the tentorium and lysis of the overlying occipital bone (Figure 2). On MRI the mass was isointense to grey matter on both T1- and T2-weighted images (WI), flow voids were noted (Figure 3, arrowheads). The lesion demonstrated mildly heterogenous, avid contrast enhancement (Figure 4). There was no restricted diffusion. The patient underwent selective intra-arterial embolization before surgical resection to minimize blood loss. Afterwards the patient received adjuvant radiotherapy.

## Comment

HPC arise from the mesenchymal spindle cells and histologically demonstrate considerable overlap with *Solitary Fibrous Tumors*. They are hence grouped in the WHO 2016 classification of tumors of the central nervous system under mesenchymal, non-meningothelial tumors, ranging from grade I to III. HPC/SFT can occur in soft tissue and intracranially. It is an exceedingly rare tumor representing only <2% of the soft tissue tumors and 0.4% of CNS tumors. On imaging CNS HPC/SFT shows vivid, heterogeneous enhancement and may exhibit a dural tail. Due to these similarities with meningioma, this lesion was previously termed ‘angioblastic meningioma’. Only later the histologic similarities with the HPC/SFT were discovered. HPC/SFT occurs more often in younger male patients, in contrast to meningioma, which is more prevalent in older, female patients. Imaging clues in favour of HPC/SFT over meningioma are lobulated, mushrooming contours; lysis of the adjacent bone; absence of calcification or hyperostosis; and edema in the adjacent brain parenchyma in most cases. MRI spectroscopy may help in differentiation as HPC/SFT can exhibit a high myo-inositol peak [1]. The treatment of choice is local resection and adjuvant radiotherapy. HPC/SFT is known to recur and produce distant metastases after long periods of time, typically in lung or bone. Our patient presented with typical imaging findings for CNS HPC/SFT, namely an extra-axial intracranial enhancing tumor with flow-voids and lysis of the underlying occipital bone. The occurrence of CNS manifestation of HPC/SFT in a patient with history of musculoskeletal HPC could be either coincidental, syndromic (unknown genetic defect), or metastatic.
